# Superior Field Emission Properties of Layered WS_2_-RGO Nanocomposites

**DOI:** 10.1038/srep03282

**Published:** 2013-11-21

**Authors:** Chandra Sekhar Rout, Padmashree D. Joshi, Ranjit V. Kashid, Dilip S. Joag, Mahendra A. More, Adam J. Simbeck, Morris Washington, Saroj K. Nayak, Dattatray J. Late

**Affiliations:** 1 School of Basic Sciences, Indian Institute of Technology, Bhubaneswar 751013, India; 2 Center for Advanced Studies in Material Science and Condensed Matter Physics, Department of Physics, University of Pune, Pune 411007, India; 3 Department of Physics, Applied Physics, and Astronomy, Rensselaer Polytechnic Institute, Troy, New York 12180, USA; 4 Physical & Materials Chemistry Division, CSIR-National Chemical Laboratory, Pashan Road, Pune 411008, India

## Abstract

We report here the field emission studies of a layered WS_2_-RGO composite at the base pressure of ~1 × 10^−8^ mbar. The turn on field required to draw a field emission current density of 1 μA/cm^2^ is found to be 3.5, 2.3 and 2 V/μm for WS_2_, RGO and the WS_2_-RGO composite respectively. The enhanced field emission behavior observed for the WS_2_-RGO nanocomposite is attributed to a high field enhancement factor of 2978, which is associated with the surface protrusions of the single-to-few layer thick sheets of the nanocomposite. The highest current density of ~800 μA/cm^2^ is drawn at an applied field of 4.1 V/μm from a few layers of the WS_2_-RGO nanocomposite. Furthermore, first-principles density functional calculations suggest that the enhanced field emission may also be due to an overalp of the electronic structures of WS_2_ and RGO, where graphene-like states are dumped in the region of the WS_2_ fundamental gap.

Following the graphene^1^,^2^,^3^,^4^ revolution, graphene analogues of other inorganic layered materials have received significant attention from the scientific community due to their interesting and useful properties as well as their direct applications in various nanoelectronic devices. Among all the layered compounds: MoS_2_^4^,^5^,^6^,^7^,^8^,^9^,^10^,^11^,^12^, MoSe_2_^13^,^14^,^15^,^16^, WS_2_^6^,^17^,^18^, WSe_2_^19^,^20^,^21^, GaS^7^,^22^,^23^, GaSe^7^,^22^,^24^, TaS_2_^25^,^26^, RhTe_2_^27^, PdTe_2_^28^ are semiconductors; h-BN^29^,^30^ and HfS_2_^31^ are insulators; NbS_2_^32^, NbSe_2_^33^, NbTe_2_^34^, and TaSe_2_^26^,^35^,^36^ are superconductors; while Bi_2_Te_3_^37^,^38^ and Bi_2_Se_3_^39^,^40^ act as topological insulators with good thermoelectric properties.

Field electron emission is the extraction of electrons from conducting/semiconducting materials via tunneling through the surface potential barrier by applying a very strong electric field of the order of 10^6^–10^7^ V/cm. Field emission has technological applications in various micro/nano-electronic devices. There is a great interest in the development of field emission based cathodes using various 1-dimesnional (1D) and 2-dimesional (2D) nanostructured materials. 1D and 2D materials such as carbon nanotubes (CNTs)^41^,^42^, ZnO^43^,^44^,^45^, LaB_6_^46^,^47^,^48^_,_ graphene^49^,^50^, reduced graphene oxide (RGO)^51^ and MoS_2_^52^ have emerged as potential field emitter candidates. 2D materials are known for their atomically thin planar structure, which is already utilized in flat technology such as flat panel field emission displays. Amongst all 2D materials, graphene, GO and layered MoS_2_ sheets have been recently explored by researchers for their field emission properties. Graphene analogues of other 2D layered materials have emerged in material science and nanotechnology due to the enriched physics and novel enhanced properties they present. There are several advantages of using 2D nanomaterials in field emission based devices, including a thickness of only a few atomic layers, high aspect ratio (the ratio of lateral size to sheet thickness), excellent electrical properties, extraordinary mechanical strength and ease of synthesis. Furthermore, the presence of edges can enhance the tunneling probability for the electrons in layered nanomaterials similar to that seen in nanotubes^41^,^42^.

The inorganic chalcogenide material WS_2_ is a naturally occurring tungstite compound formed by 2D covalently bonded S-W-S layers separated by a van der Waals gap. Weak van der Waals interactions also hold the adjacent sulphur sheets together with a layer sequence S-W-S^6^,^17^,^18^,^53^. WS_2_ possesses hexagonal crystal structure with space group *P*63/*mmc* and each WS_2_ monolayer contains an individual layer of W atoms with 6-fold coordination symmetry, which are then hexagonally packed between two trigonal atomic layers of S atoms^6^,^17^,^18^,^53^. The WS_2_ material has attracted attention for diverse applications in future nanoelectronic devices because of its 2D layered structure and direct-band gap^6^,^17^,^18^. Whereas bulk WS_2_ has an indirect band gap of 1.35 eV, when it is thinned to a single layer it becomes direct band gap semiconductor with a gap of 2.05 eV^17^,^18^.

We have recently reported field emission properties of layered MoS_2_ sheets exhibiting a turn on field of 3.5 V/μm to draw a current density of 10 μA/cm^2^ 52. This has generated interest in field emission studies of other transition metal dichalcogenides such as WS_2_. Furthermore, in an attempt to enhance the field emission properties of WS_2_, we have prepared a composite of WS_2_ on RGO by a low-temperature hydrothermal method. We report here for the first time field emission studies on a layered WS_2_ and WS_2_-RGO nanocomposite, where the RGO supported system exhibits superior field emission. We relate this superior performance to ehanced electric fields at the sheet edges and surface protrusions in the WS_2_-RGO composite. In addition, first-principles density functional theory (DFT) calculations show that the enhanced emission may also be due in part to the overlapping nature of the electronic structure of the composite system.

## Results

**Figure 1:**
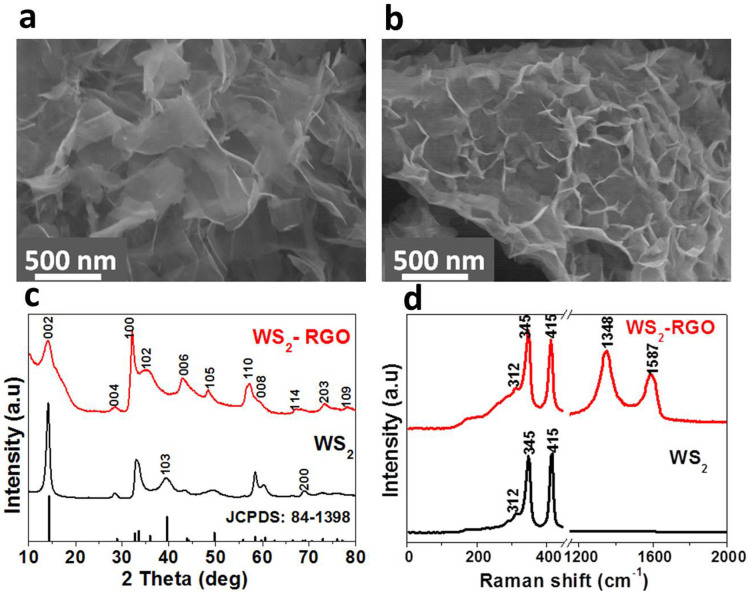
Field emission scanning electron microscopy (FE-SEM) images of the (a) WS_2_ and (b) WS_2_-RGO nanocomposite emitters. (c) XRD patterns of WS_2_ sheets and WS_2_-RGO nanocomposite. (d) Raman spectra of WS_2_ and WS_2_-RGO nanocomposite.

Fig. 1 shows a field emission scanning electron microscope (FE-SEM) image of single-layer to a few-layered WS_2_ sheets (Fig. 1a) and WS_2_-RGO nanocomposite sheets deposited on a Si substrate (Fig. 1b). The FE-SEM images reveal that the thickness of stacked WS_2_ sheets is ~1–5 nm and their length is in the range of ~1–3 μm. These images also reveal that the Si substrate was completely covered with WS_2_ sheets and that the sheets possess a rough morphology along with vertical aligmnement (see Fig. S1, Supplementary Information). The FE-SEM images of composite WS_2_-RGO exhibit a large number of protruding edges on the surface as compared to WS_2_ and RGO sheets (see Supplementary Information, Fig. S1). Fig. 1c shows the typical X-Ray diffraction (XRD) pattern of WS_2_ sheets and the WS_2_-RGO nanocomposite. XRD analysis of the WS_2_ sheets and WS_2_-RGO nanocomposite shows high crystalline hexagonal structure [Powder diffraction file (PDF) no. 84-1398] without any other impurities. The XRD data of WS_2_ sheets shows the direction of sheet growth is along the (002) direction. The XRD pattern of the WS_2_-RGO composite shows a broad (002) peak and a more intense (100) peak as compared to the WS_2_ sheets. The broadness of the (002) peak indicates both smaller size and fewer layers for the WS_2_ sheets. Also, it confirms the growth of a large number of protrusion edges along the (100) direction on RGO. Raman spectroscopy reveals the characteristic peaks of WS_2_ in the 200–500 cm^−1^ range and the D (1348 cm^−1^) and G (1587 cm^−1^) bands of RGO in the WS_2_-RGO composite (Fig. 1d). In both the WS_2_ sheets and the WS_2_-RGO composite three bands are observed at 312, 345 and 415 cm^−1^ which corresponds to the E_1g_, E_2g_^1^ and A_1g_ modes, respectively^54^,^55^.

**Figure 2:**
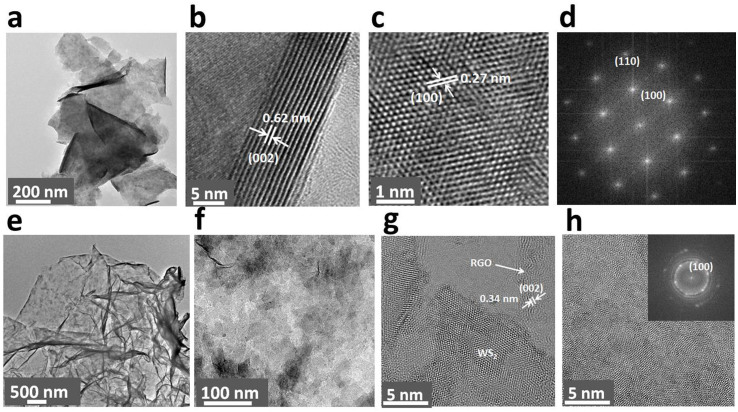
TEM analysis of WS_2_ sheets: (a) low magnfication image, (b) high resutlon image showing the multilayer nature of the sheets, (c) high resultion image of the sheets showing the hexagonal structure of WS_2_ and (d) fast Fourier transform of the electron diffraction pattern of a few layers of WS_2_. TEM analysis of WS_2_-RGO sheets: (e, f) TEM images and (g, h) HRTEM images showing (002) lattice planes of RGO. Inset of (h) is a selected area for electron diffraction patterning of a few layers of WS_2_.

Transmission Elecron micrscopy (TEM) analysis demonstrates the formation of single crystalline, few-layered WS_2_ sheets (Fig. 2 and Supplementary Information, Fig. S2). The high-resultion Transmission Elecron micrsocopy (HRTEM) image revealed stacking of WS_2_ (002) layers with an Interplanar spacing of 0.62 nm and periodic arrays of (100) planes with a spacing of 0.27 nm (Fig. 2c). In the planar orientation, lattice fringes along (100) and (110) planes of the hexagonal WS_2_ are clearly observed (Fig. 2c, d). Fig. 2e, f show TEM images of WS_2_-RGO sheets, which indicate uniform coverage of WS_2_ on RGO. HRTEM analysis reveals epitaxial growth of thin layered, hexagonal WS_2_ on RGO (Fig. 2g, h and Supplementary Information S3). HRTEM analysis near the edge of the WS_2_ sheets shows an interlayer spacing of ~0.34 nm, which confirms the growth of WS_2_ from that of the graphene sheet (Fig. 2c and Supplementary Information, Fig. S3). Since GO sheets exhibit enormously active edges and functional groups on their basal plane, they act as a novel substrate for the nucleation and subsequent growth of WS_2_. Hence during hydrothermal reaction with a GO solution, the tungsten precursor was reduced to form WS_2_ on GO and GO transformed to RGO.

## Discussion

As described above, the hybrid nanostructures of 2D materials can be controllably prepared by the simple hydrothermal method. The hybrid nanostructures consisting of two 2D materials have numerous sharp edges and a huge proportion of nano-protrusions. Due to the unique morphologies, the hybrid nanostructures should have enhanced field emission properties. For comparison, we also show the field emission properties of pure RGO and WS_2_ sheets.

The Fowler-Nordehim (F-N) equation for field emitters deposited on flat substrates has been suitably modified to yield an equation in terms of current density (*J*) and the applied electric field (*E* = *V/d*, where *V* is the voltage applied between the flat cathode and the anode screen, and *d* is their separation). The modified F-N equation is as follows^56^,^57^, 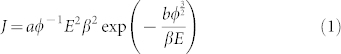
where *a* and *b* are constants (*a* = 1.54 × 10^−6^ AeV V^−2^, *b* = 6.83 eV^−3/2^ Vnm^−1^), *J* is the current density, *E* is the local electric field (surface field) and *β* is the local electric field enhancment factor.

**Figure 3:**
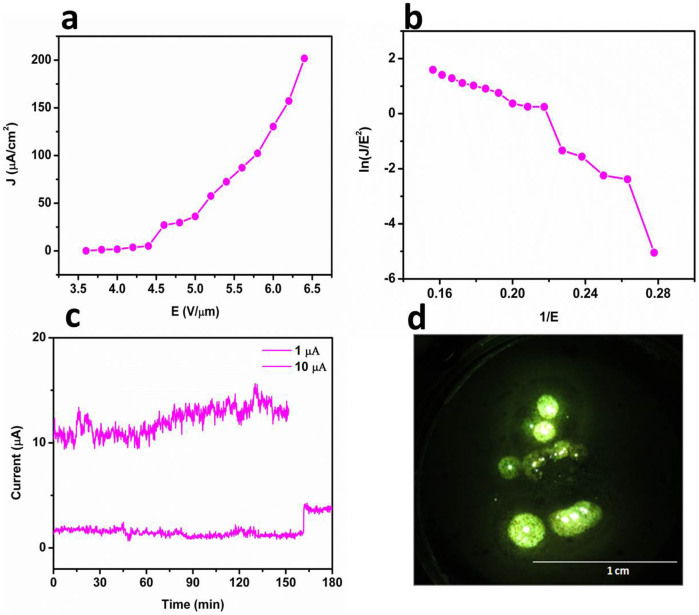
Field emission from few-layered WS_2_ sheets. (a) Applied electrical field as a function of emission current density. (b) F-N plot showing non-linear behaviour indicating emission current from the semiconducting emitter. (c) Long term field emission current stabilty indiacting fairly stable emission current. (d) Field emission pattern taken during the long term stability study of the emitter.

**Figure 4:**
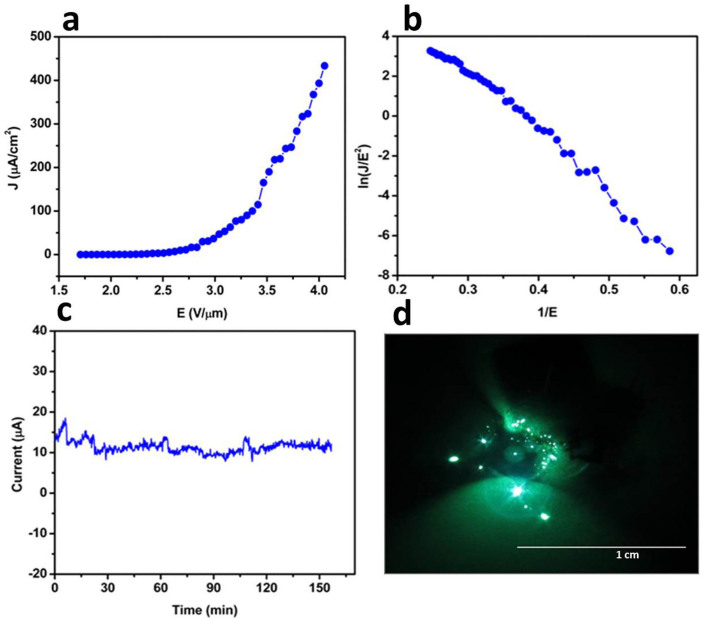
Field emission from RGO sheets. (a) Applied electrical field as a function of emission current density. (b) F-N plot showing linear behaviour indicating emission current from the metallic emitter. (c) Long term field emission current stabilty (at 10 μA) indiacting fairly stable emission current. (d) Field emission pattern taken during the long term stability study of the emitter.

The plot of the field emission current density *J* versus applied electric field *E* for WS_2_ is shown in Fig. 3a. The F-N plot [a plot of *ln (J/E*^2^*)* versus *1/E*] for the WS_2_ sheets is shown in Fig. 3b with a calculated field enhancement factor of ~1182 (calculated from slope of the linear region of F-N plot). The F-N plot for the WS_2_ field emitter is nearly linear and shows a tendency for saturation at high electric fields. Fig. 3c shows the typical long term current stability from a WS_2_ nanosheet field emitter. Fig. 3d shows the typical field emission micrograph of the WS_2_ nanosheet field emitter recorded at a current density of 50 μA/cm^2^. The field emission from RGO sheets is shown in Fig. 4a as a function of applied electrical field versus emission current density. Fig. 4b shows the corresponding F-N plot showing linear behaviour. Fig. 4c shows the long term field emission current stabilty for RGO sheets, indiacting a stable emission current. Fig. 4d shows the field emission pattern for RGO sheets taken during long-term current stability measurments of the emitter.

**Figure 5:**
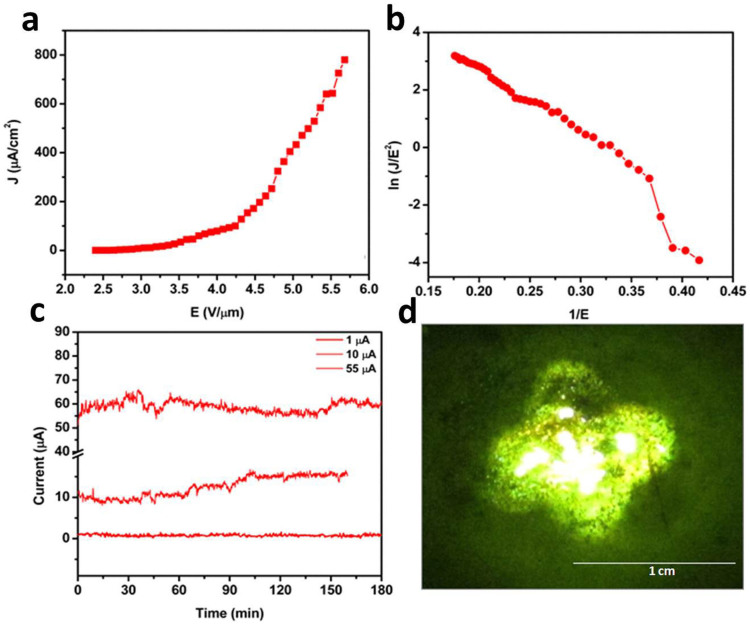
Field emission studies of few-layered WS_2_-RGO nanosheets. (a) Applied electric field *vs.* field emission current density. (b) F-N plot showing non-linear behaviour typical of the semiconducting emitter. (c) Long term field emission current stabilty at 1, 10 and 50 μA indiacting fluctuations in stability due to adsorption and desorption of gas molcules. (d) Field emission pattern taken during the long term stability study of the emitter.

Fig. 5a shows a *J-E* plot for the WS_2_-RGO nanocomposite. Fig. 5b depicts the corresponding F-N plot for the WS_2_-RGO nanocomposite with a field enhancement factor of 2994. Fig. 5c shows the long term current stability measurements for WS_2_-RGO nanocomposites. Fig. 5d shows the typical field emission micrograph of WS_2_-RGO sheets recored at a current density 50 μA/cm^2^. The field emission micrograph of the WS_2_-RGO composite consists of a large number of tiny bright spots and more uniform emission as compared to the WS_2_ and RGO field emitter.

The field enhancement factor can provide a quantitative idea of the degree of enhancement of the electric field at the emitter (WS_2_ and WS_2_-RGO) sheet edges due to their nanometric dimension. In the present case, the field enhancement factor is calculated from the slope of the F-N plots using 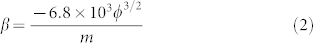
where *β* represnts the field enhacment factor, *m* is slope of F-N plot and *ϕ* is the workfunction of the emitter, which is determined from density functional theory (DFT) calculations to be 5.89 eV for WS_2_ and 4.48 eV for RGO (see discussion below and Computational Methods section for details). The field enhacment factor values calculated from equation (2) are found to be 2468, 2619 and 2978 for WS_2_, RGO and the WS_2_-RGO nanocomposite field emitters, respectively. Table 1 in the Supplementary Information shows a comparison of electric field values required to draw an emission current density of 1 μA/cm^2^, 10 μA/cm^2^ and 100 μA/cm^2^ (See. Supplementary Information).The observed turn on and threshold values for the WS_2_-RGO nanocomposite are significantly lower than that of the few-layered WS_2_ field emitter. The low turn on values in the case of WS_2_-RGO are attributed to the atomically sharp edges of the WS_2_-RGO nanocomposite sheets, which are reflected by the high value of the field ehnacment factor as compared to the WS_2_ field emitter. This can be explained on the basis of FE-SEM imaging which shows a higher concentration of protruding edges in the case of the WS_2_-RGO composite than in case of WS_2_ sheets_._ Also, the observed field emission image for WS_2_-RGO clearly depicts a higher density of emission spots for the emitter, corroborating with the estimated values of *β* as explained above. Furthermore, the DFT calculations show that in addition to the surface protrusions and edge effects, the enhanced field emission may also be partly attributed to the overlapping electronic structure of the composite.

**Figure 6:**
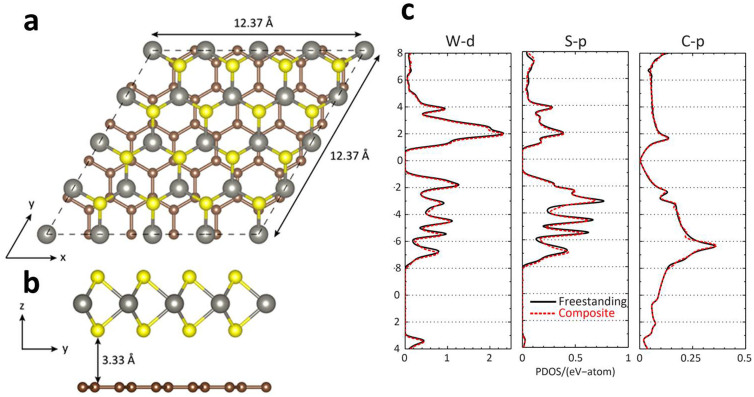
(a) Top and (b) side views of the composite WS_2_-graphene system. The system contains a total of 98 atoms: 32 S (yellow/light), 16 W (grey), and 50 C (brown/dark). The lattice mismatch between the two monolayers has been treated by straining both WS_2_ and graphene. (c) Select partial density of states (PDOS) of freestanding (black, solid) and composite (red/grey, dashed) systems. From left to right: W *d*-states, S *p*-states, and C *p*-states. The presence of the graphene substrate leaves the WS_2_ states intact while adding states in the WS_2_ fundatmental gap region. The zero of energy for the free standing WS_2_ monolayer has been taken as half the band gap.

The geometry of the composite system is featured in Fig. 6a, b. As mentioned in the Computational Methods section, for simplicity, the composite system is taken as WS_2_ atop graphene, and in order to match the two lattices the strain is shared between the two monolayers. The strained WS_2_ lattice constant is taken as 3.09 Å (−1.18%) and graphene as 2.48 Å (+1.21%). Here we have defined the strain relative to the theoretically predicted lattice constants. This relatively small amount of strain has minor effects on the geometry and electronic structure of WS_2_ and graphene. For example, optimization of the pristine WS_2_ unit cell yields bond lengths of W-S = 2.39 Å and S-S = 3.12 Å, in agreement with ref. 58, whereas in the strained cell W-S = 2.38 Å and S-S = 3.15 Å. Quadrupling (Quintupling) the WS_2_ (graphene) lattice then gives the supercell lattice constant of 12.37 Å for the composite system. To combine the two monolayers, WS_2_ is simply placed atop graphene at a separation of 3.33 Å (Fig. 6b). Note that rotational and other lattice mismatch effects have not been considered in this work, and are not suspected to alter the conclusions.

From our self-consistent energy calculations the work function *φ* of the free-standing systems can be calculated using 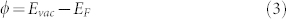
where *E_vac_* is the converged electrostatic potential in the vacuum region and *E_F_* is the Fermi energy. The work functions of strained WS_2_ *φ_WS_*_2_ and graphene *φ_G_* are computed using Equation 3. Our methodology is verified by the calculation of the work function for pristine graphene *φ_G_* = 4.48 eV, which is in perfect agreement with ref. 59. For the strained WS_2_ monolayer it is found that *φ_WS_*_2_ = 5.89 eV, whereas for strained graphene the work function, compared to WS2, is predicted to be significantly smaller: *φ_G_* = 4.58 eV. The change in the respective work functions when the composite is formed is negligible.

The projced density of states (PDOS) analysis is featured in Fig. 6c, where only the states which give major contributions to the bands near the Fermi energy are shown, *i.e.* W *d*-states, S *p*-states, and C *p*-states (Fig. 6c, left, middle, and right, respectively). Note that for the freestanding WS_2_ system the DOS has been centered on the band gap, whereas for the composite system the Fermi energy is determined by graphene. The presence of the graphene substrate essentially has no appreciable effect on the WS_2_ DOS, and vice-versa, besides dumping states in the band gap region of WS_2_. This addition of states, combined with the unappreciable change in work function, suggests that the composite system takes advantage of the *best of both worlds* in terms of the field emission properties of WS_2_ and graphene. When an electric field is applied to the system electrons will first be removed from states nearest the Fermi energy, which in the composite system are due to graphene. Although the DOS is very low near *E_F_* for graphene the work function is small, compared to WS_2_, so that electrons are able to escape at a lower applied field. Eventually though, after continually increasing the bias, electrons will be also be emitted by WS_2_. The work function of WS_2_ is larger than that of graphene, by 1.31 eV, but now the DOS has dramatically increased and there are more electrons available for emission. Therefore the combined effect of the relative DOS and relative work functions of WS_2_ and graphene also may play a role in the experimentally observed enhanced field emission.

In addition to increased performance, the applications of field emitters also require emission current stability, so it is a decisive and important parameter in the fabrication of field emission based nanoelectronic devices. Fig. 3c and Fig. 5c show the field emission current stability traces for WS_2_ and the WS_2_-RGO nanocomposite field emitters at different preset values for a sampling interval of 10 seconds recorded over a period of 3 hours. It has been observed that both WS_2_ and WS_2_-RGO show *spike* type fluctuations in the field emission current. The main cause of these *spike*-like fluctuations in emission current is adsorption/desorption and ion bombardment due to residual gas molecules^8^. Thus, during adsorption/desorption events the local work function varies slightly, depending upon the nature of the molecule (either electropositive or electronegative), on the emitter surface. The ion bombardment with residual gas molecules due to the presence of high electrostatic fields results in mechanical damage, further causing creation and destruction of emission sites, which in turn causes the fluctuations in the field emission current.

In summary, the field emission properties of WS_2_ and WS_2_-RGO have been investigated at the base pressure of ~1 × 10^−8^ mbar. The turn on field required to draw a current density of 1 μA/cm^2^ is found to be 3.5 V/μm and 2 V/μm for WS_2_ and the WS_2_-RGO composite, respectively. Enhanced field emission behavior is observed for WS_2_-RGO due to a high field enhancement factor associated with surface protrusions. In addition, the DFT results show that the enhanced field emission may be compounded by the overlapping electronic structures of WS_2_ and RGO. Owing to the low turn on field and planar (sheet-like) structure morphology, the WS_2_-RGO emitter can be utilized for new generation vacuum microelectronics/nanoelectronics and flat panel display applications.

## Methods

### The preparation of few layered WS_2_ sheets

WS_2_ sheets were synthesized by a one-step hydrothermal reaction. In a typical experiment, 3 mM WCl_6_ (Sigma-aldrich, 99.98%) and 15 mM thioacetamide (C_2_H_5_NS, Sigma-Aldrich, ≥99%) were dissolved in 40 mL DI water and stirred for 1 hour at room temperature by using a magnetic stirrer. The solution was transferred to a 50 mL stainless steel autoclave, heated up to 265°C and kept for 24 hours. After cooling naturally, the product was filtered, washed with DI water and dried in vacuum at 60°C for 6 hours.

### The preparation of WS_2_-RGO composite

The WS_2_-RGO composite was synthesized by the same hydrothermal reaction condition as that for WS_2_ sheets. 8 mL of 5 mg/mL GO solution (see Supplementary Information) was added to the mixture of WCl_6_ and thioacetamide and the total volume of the solution was maintained at 40 mL. The same processes mentioned for WS_2_ sheets were followed. During the hydrothermal process, smaller size WS_2_ sheets were epitaxially formed on GO and subsequently GO transformed to RGO (see Supplementary Information, Scheme 1). Carbon content in the final product was 3 wt%, which was confirmed by elemental analysis.

### Materials characterization

The samples were characterized with X-ray diffraction equipped with the following: Ni filtered Cu Kα radiation (40 kV, 100 mA, *λ* = 0.15418 nm), field emission scanning electron microscopy and high resolution transmission electron microscopy. The samples were also characterzied by a Micro Raman spectrometer with a laser excitation wavelength of 532 nm.

### Field emission

The field emission studies of few-layered WS_2_/Si, RGO/Si and WS_2_-RGO/Si nanocomposite were investigated independently in an ultra high vacuum (UHV) chamber at the base pressure of ~1 × 10^−8^ mbar. The UHV chamber is equipped with a rotary backed turbo molecular pump, sputter ion pump and titanium sublimation pump. For achieving base pressure of ~1 × 10^−8^ mbar, the chamber was baked at 200°C for 12 hours. The field emission studies were carried out in close proximity setup, which was mounted in the UHV chamber. The close proximity setup consisted of specimens (WS_2_/Si, WS_2_-RGO/Si independently) acting as the cathode and a copper rod as the anode. Inter-electrode separation could be varied from 500 μm to 1500 μm using an insulating alumina spacer. The field emission current (I) versus applied voltage (V) was measured using Keithley 6514 electrometer and Spellman high voltage DC power supply. The field emission current stability was investigated using a computer controlled data acquisition system with a sampling interval of 10 seconds. The field emission micrographs were seen on a transparent ITO coated glass with a phosphor screen (anode) and were recorded using a digital camera (Canon SX150IS).

### Computational methods

To model the WS_2_-RGO system we consider the composite system of a monolayer of WS_2_ deposited on graphene (Fig. 6a, b) within first-principles density functional theory (DFT). The DFT based Vienna *ab intio* simulation package (VASP)^60^,^61^,^62^,^63^ was employed with projector-augmented wave (PAW) pseudopotentials^64^,^65^ to describe the electron-ion interaction. The exchange-correlation energy is described using the local density approximation (LDA)^66^ and it was found that the generalized gradient approximation (GGA)^67^,^68^ produces similar results. In order to match the WS_2_ lattice to that of graphene, both monolayers are strained between 1–2%. To begin, the strained unit cell geometries are optimized until the forces on each atom are less than 0.01 eV/Å. For WS_2_ (graphene), a plane-wave basis energy cutoff of 400 eV (600 eV), a 12 × 12 × 1 (18 × 18 × 1) Monkhorst-Pack k-point sampling^69^, and at least 12.5 Å (10 Å) of vacuum is necessary to see convergence in the total energy on the order of 1 meV per atom for the strained unit cells. Next, the strained unit cells are expanded and combined to form the composite supercell: WS_2_ by 4× and graphene by 5× for a total of 98 atoms (32 S, 16 W, and 50 C). WS_2_ is placed atop graphene at a fixed distance of 3.33 Å (Fig. 6b), representative of the van der Waals interaction between the two monolayers. Note that any further optimization of the geometry of the supercell does not affect the reported results. The strained supercell for the composite system employs similar parameters, except the k-point sampling is reduced to 6 × 6 × 1. The energy of both the unit/super-cell is relaxed until differences in total energy are less than 10^−4^ eV and Gaussian smearing with a width of 0.05 eV is used to describe the partial occupancies of the orbitals. From our self-consistent energy calculations the work function φ of the free-standing systems can be calculated using Equation (3) above. Note that in order for the electrostatic potential to converge smoothly in the vacuum region for the composite system, dipole corrections must be included to treat the dipole interactions which arise between periodic images in the asymmetric slab model^70^. Finally, the results are also accompanied by a partial density of states (PDOS) analysis for the dominant states near the Fermi energy (Fig. 6c) in order to interpret the field emission properties of WS_2_-RGO.

## Author Contributions

C.S.R., D.J.L. and S.K.N. designed experiment. C.S.R., P.D.J., R.V.K. carried out experiment. A.J.S. performed computational calculation. C.S.R., D.S.J., M.A.M., M.W., S.K.N. and D.J.L. carried out data analysis and co-wrote the manuscript. All authors reviewed the manuscript.

## Supplementary Material

Supplementary Information Supplementary information
