# Supplementary material for: Superior Field Emission Properties of Layered WS2-RGO Nanocomposites
Source: Sci Rep. 2013 Nov 21;3:3282. doi: 10.1038/srep03282 (PMC3836036; doi:10.1038/srep03282)
Supplement: Supplementary Information [file srep03282-s1.pdf]

Supplementary Information

# Superior Field Emission Properties of Layered $WS_2$-RGO Nanocomposites

Chandra Sekhar Rout,[1*] Padmashree D. Joshi,[2] Ranjit V. Kashid,[2] Dilip S. Joag,[2] Mahendra A. More,[2] Adam J. Simbeck,[3] Morris Washington,[3] Saroj K. Nayak,[1, 3*] Dattatray J. Late[4*]

[1]School of Basic Sciences, Indian Institute of Technology Bhubaneswar, Bhubaneswar 751013, India

[2]Center for Advanced Studies in Material Science and Condensed Matter Physics, Department of Physics, University of Pune, Pune 411007, India.

[3]Department of Physics, Applied Physics, and Astronomy, Rensselaer Polytechnic Institute, Troy, New York 12180, USA

[4]Physical & Materials Chemistry Division, National Chemical Laboratory, Pashan Road, Pune 411008, India

**S1. Synthesis of GO:**

Graphene oxide (GO) synthesis was performed using modified Hummers method [1, 2]. In the general synthesis process, concentrated $H_2SO_4$ (50 mL) was added to graphite powder (SP-1, Bay carbon), $K_2S_2O_8$(1 g), and $P_2O_5$(1 g) in a round-bottomed flask and heated at 80 $^oC$. The obtained mixture was stirred using a magnetic stirrer and maintained at a temperature of 0 °C in ice bath. Potassium permanganate ($KMnO_4$, 6g) was slowly added to the solution and stirred for 2hrs. The reaction was terminated by addition of excess amount of distilled water and 5 mL $H_2O_2$ solution. The mixture was filtered and washed with excessHCl.The resultinggraphite oxide was suspended in distilled water again, followed by dialysis (Dialysis membrane: Spectrum Laboratories, MWCO-12-14,000) to remove excess HCl.The graphite oxidewas exfoliated to give ~5 mg/mL GO solution by ultrasonication. After exfoliation, the solution was centrifuged at 3500 rpm for 10 min to remove the non-exfoliated graphite oxide and the top supernatant GO solution was used for hydrothermal reaction. Presence of oxygen functional groups makes the few-layered GO sheets highly hydrophilic and a stable dispersion was obtained.

**S2. Schematic showing hydrothermal synthesis process of $WS_2$-RGO composite**

**Scheme 1:**

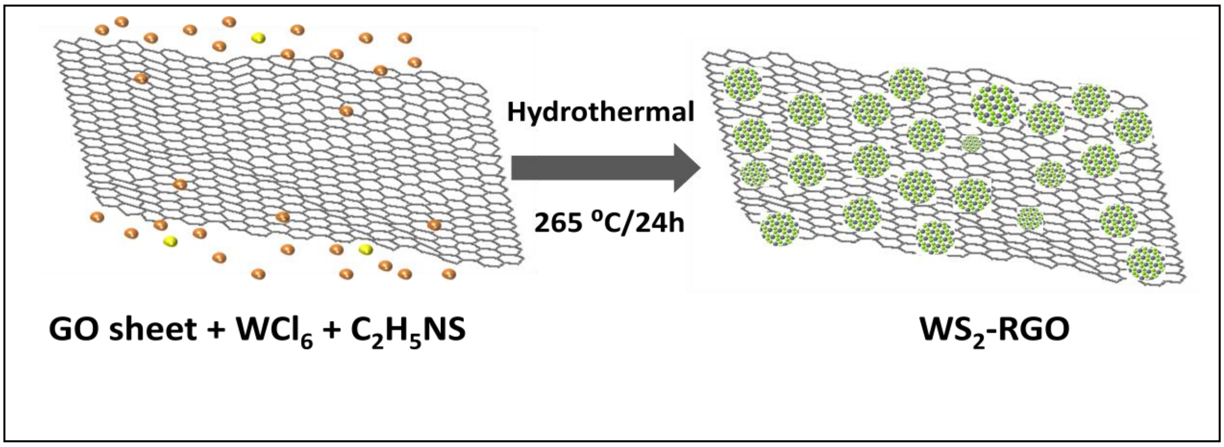

**Figure S1**: FESEM image showing coverage of (a) $WS_2$ sheets (b) $WS_2$-RGO composites on Si and overall sheets has rough morphology along with vertical aligmnement. (c, d) FESEM images of RGO.

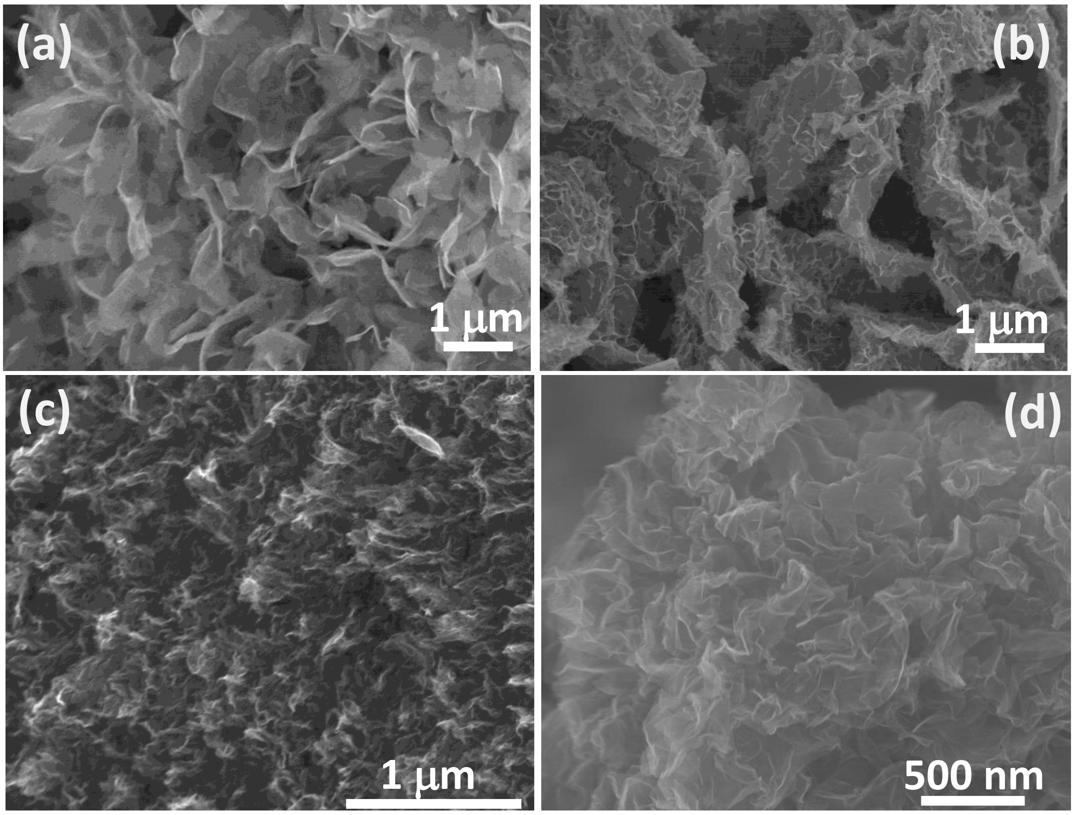

**Figure S2**: HRTEM images of a $WS_2$ sheet consisting of two layers with single-crystalline hexagonal structure.

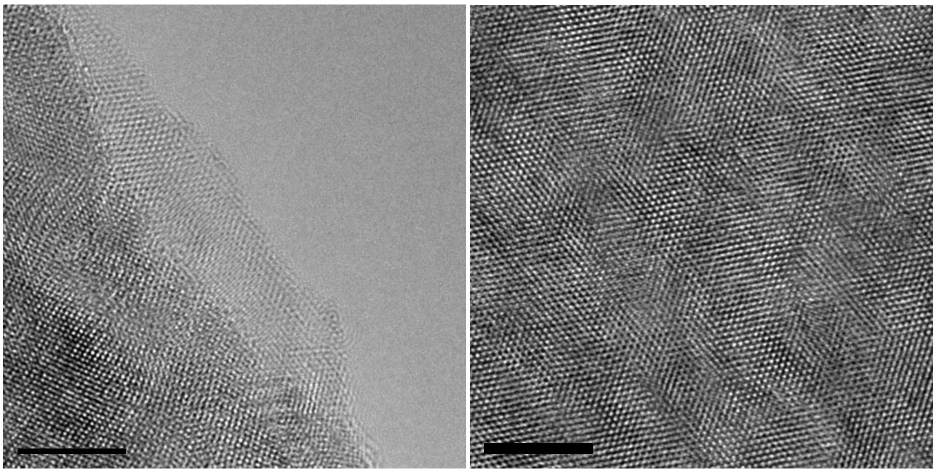

**Figure S3**: HRTEM of $WS_2$-RGO showing epitaxial growth of crystalline $WS_2$ on RGO.

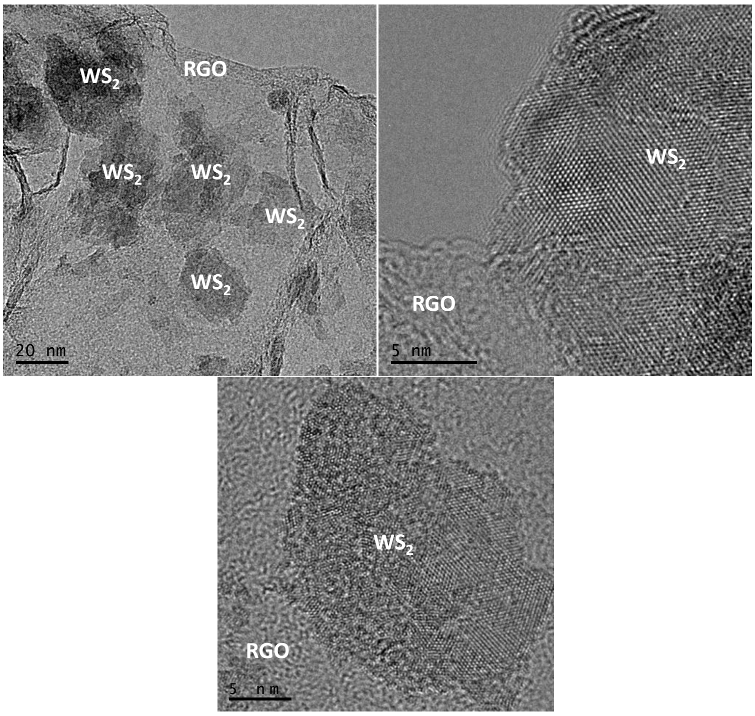

**Table 1:** Comparasion of turn on field and field enhancement factor for $WS_2$ sheets and the $WS_2$-RGO nanocomposite field emitters.

| **Field emitter** (for a cathode-anode sepration of 1000 µm) | **Turn on field for emission density J (1µA/cm$^2$)** | **Turn on field for emission density J (10µA/cm2)** | **Field enhancement factor β** |
|---|---|---|---|
| $WS_2$ sheets | 3.5 V/µm | 4.6 V/µm | 2468 |
| RGO sheets | 2.3 V/µm | 2.7 V/µm | 2619 |
| $WS_2$-RGO sheets | 2.0 V/µm | 2.5 V/µm | 2978 |

**References**

[1] Kovtyukhova, N.I. *et al.* Layer-by-layer assembly of ultrathin composite films from micron-sized graphite oxide sheets and polycations, *Chem. Mater.***11**, 771-778 (1999).
[2] Park, S. *et al.*Aqueous suspension and characterization of chemically modified graphene sheets, *Chem. Mater.***20**, 6592-6594 (2008).
